# Effects of miR-19b knockdown on the cardiac differentiation of P19 mouse embryonic carcinoma cells

**DOI:** 10.3892/mmr.2014.3037

**Published:** 2014-12-03

**Authors:** XUEHUA LIU, LIMING YANG, HUIYAN WANG, GUOFENG XU, SHASHA ZHU, MENGMENG LI, XIAOSHAN HU, JINGAI ZHU, CHUN ZHU, JING XU, SHUPING HAN, ZHANGBIN YU

**Affiliations:** 1 State Key Laboratory of Reproductive Medicine, Department of Pediatrics, Nanjing Maternity and Child Health Care Hospital Affiliated to Nanjing Medical University, Nanjing, Jiangsu 210004, P.R. China; 2 Department of Pediatrics, Changzhou Maternity and Child Health Care Hospital, Changzhou, Jiangsu 213003, P.R. China

**Keywords:** miR-19b knockdown, apoptosis, P19 cells, congenital heart disease

## Abstract

MicroRNA-19b (miR-19b) is part of the miR-17–92 cluster which is associated with cardiac development. It has previously been reported that the overexpression of miR-19b increases proliferation, inhibits apoptosis and promotes differentiation of embryonic carcinoma cells (P19 cells). The aim of the current study was to investigate the effects of miR-19b knockdown on the proliferation, apoptosis, differentiation and regulation of the Wnt/β-catenin signaling pathway in P19 cells. P19 cells were transfected with an miR-19b knockdown plasmid or an empty vector. MiR-19b knockdown or vector control stable cell lines were selected using puromycin. Cell Counting kit-8 and flow cytometry were used to analyze the levels of cellular proliferation, cell cycle progression and the levels of apoptosis, respectively. Caspase-3 activity and mitochondrial function assays were also used to analyze apoptosis. An inverted microscope was used to observe the morphological changes of P19 cells during differentiation. Reverse transcription-quantitative polymerase chain reaction and western blot analysis were used to detect P19 cell differentiation markers and Wnt/β-catenin signaling pathway-related genes and their corresponding proteins. The results demonstrated that miR-19b knockdown inhibited the proliferation and apoptosis of P19 cells. However, the levels of expression of Wnt and β-catenin increased. MiR-19b knockdown activated the Wnt/β-catenin signaling pathway, which may regulate cardiomyocyte differentiation. The results of this study indicate that miR-19b is a novel therapeutic target for cardiovascular diseases and provide insight into the mechanisms underlying congenital heart diseases.

## Introduction

Congenital heart defects (CHD), which include malformations of the heart or great vessels, are the most common group of major birth defects, with an incidence of 5–8 per 1,000 live births (1). MicroRNAs (miRNAs) that contribute to cardiac development have been identified and can be used as novel biomarkers and therapeutic targets for CHD (2), as previously demonstrated with non-small cell lung cancer (3). MicroRNA-19b (miR-19b) is part of the miR-17–92 cluster, which encodes miR-17, miR-18a, miR-19a, miR-19b, miR-20a and miR-92a-1. The miR-17–92 cluster is required to induce cardiomyocyte proliferation in postnatal and adult hearts (4). A number of studies have shown that the miR-17–92 cluster contributes to the development of the heart, lungs, blood vessels and immune system (5). A previous study has observed specific changes in miRNA abundance and activity in a broad range of human aging models and suggested the use of miR-17, miR-19b, miR-20a and miR-106 as novel biomarkers of cellular aging (6).

P19 cells, isolated from an experimental embryo-derived mouse teratocarcinoma, differentiate into embryonic myocardial cells when exposed to dimethylsulfoxide (DMSO) (7). Therefore, they can be used to investigate cardiac-specific transcription factors and upstream signaling pathways during cardiac cell differentiation (8–10). In addition, P19 cells are an excellent model system for studying the regulation of myocardial electrophysiological differentiation at the molecular and functional levels (11).

The Wnt signaling pathway performs a number of functions during cardiogenesis (12). Early activation of Wnt/β-catenin signaling promotes cardiac differentiation in zebrafish embryos and mouse embryonic stem cells. Activation of Wnt/β-catenin at later stages results in the repression of cardiac differentiation (13). However, whether miR-19b knockdown affects the cardiac lineage commitment and differentiation through Wnt/β-catenin signaling remains to be determined.

As P19 cells can differentiate into cardiomyocytes, the present study investigated the underlying mechanisms of heart development by analyzing the proliferation, apoptosis and differentiation of P19 miR-19b-knockdown cells.

## Materials and methods

### Cell culture and induction of differentiation

P19 cells were obtained from the American Type Culture Collection (ATCC, Manassas, VA, USA). The cells were cultured in modified Eagle’s medium (α-MEM; Gibco-BRL, Grand Island, NY, USA) containing 10% fetal bovine serum (FBS; Gibco-BRL), 100 mg/ml streptomycin and 100 U/ml penicillin in a 5% CO_2_ atmosphere at 37°C. To induce cardiac differentiation, the cells were cultivated in 10 ml α-MEM supplemented with 10% FBS, 100 U/ml penicillin, 100 mg/ml streptomycin and 1% DMSO (Sigma, St. Louis, MO, USA) in 10-cm bacterial dishes in a 5% CO_2_ atmosphere at 37°C from days 0 to 4. On day 4, the embryoid bodies were transferred to 6-cm cell culture flasks with complete medium and cultured for an additional 8 days. Cells were harvested on differentiation days 0, 4, 8, 10 and 12. Morphological changes in the P19 cells were examined under an inverted microscope (Nikon Eclipse TE300; Nikon, Tokyo, Japan) equipped with phase-contrast objectives and a digital camera (E4500; Nikon). To investigate the differentiation process in P19 cells, quantitative polymerase chain reaction (qPCR) was used to identify the expression levels of cardiac troponin T (cTnT), GATA4 and NKX2.5 during differentiation.

### MiRNA transfection and establishment of stable cell lines

Lipofectamine 2000 was used to transfect the plasmids (pGLV3/H1/eGFP/Puro-miR-19b-3p-inhibitor sponge and pGLV3/H1/eGFP/Puro-miR-vector; GenePharma, Shanghai, China) into P19 cells. Puromycin (Invitrogen, Carlsbad, CA, USA), which kills untransduced cells upon addition of the minimum concentration, was used to select stably transduced cells.

### CCK-8 assay

Cell Counting kit-8 (CCK-8; Dojindo, Kumamoto, Japan) was used to assess cell growth according to the manufacturer’s instructions. The stable cell lines which were established with the miR-19b silencing expression plasmid or vector were seeded in 96-well plates and maintained in α-MEM supplemented with 10% FBS, 100 U/ml penicillin and 100 mg/ml streptomycin for seven consecutive days. In brief, the CCK-8 solution (10% of the medium, 10 μl) was added to each well and incubated for 1 h prior to analysis with a microplate reader (DNM-9602, Beijing Perlong Medical Instrument Ltd, Beijing, China) with the absorbance measured at a wavelength of 450 nm. The results were plotted as the mean ± standard deviation of three separate experiments, with three determinations per experiment for each experimental condition.

### Cell cycle assay

MiR-19b silenced or vector control stable P19 cells were plated in α-MEM with 10% FBS, 100 U/ml penicillin and 100 mg/ml streptomycin. The cells were serum deprived for 24 h to synchronize them and, following replacement of the starvation medium with complete medium, were harvested using trypsin/EDTA, washed twice with phosphate-buffered saline (PBS), fixed in 70% ethanol at −20°C overnight and then stained with 500 ml propidium iodide (PI) solution (100 mg/ml RNase and 50 mg/ml PI in 1X PBS). Cell cycle analysis was initiated at multiple time points (0, 8, 16, 24 and 32 h). BD FACScan and Cell Quest software (BD Biosciences, San Jose, CA, USA) were used to analyze the labeled cultured cells.

### Reverse transcription-quantitative polymerase chain reaction (RT-qPCR)

Total RNA was extracted from P19 cells using the TRIzol reagent and cDNA was synthesized from 1 μg of total RNA using the High Capacity cDNA Reverse Transcription kit. qPCR (Taqman method) was performed in a Sequence Detection System 7500 (Applied Biosystems Life Technologies, Foster City, CA, USA) according to the manufacturer’s instructions. All other materials including the Taqman dye and probes were obtained from Invitrogen. Briefly, the samples were incubated at 25°C for 10 min for the initial denatuation and subsequently subjected to 40 cycles of PCR, each consisting of 37°C for 120 min and 85°C for 5 min. The β-actin gene was used as a reference to obtain the relative fold change.

### Flow cytometry

Cells were cultured in serum-deprived α-MEM for 24 h to induce apoptosis. Cells were harvested using trypsin/EDTA, washed with PBS, resuspended in 1 ml binding buffer, and stained with 10 μl Annexin V-fluorescein isothiocyanate (V-FITC) and 10 μl propidium iodide (PI) at room temperature for 15 min. Flow cytometry (Carl Zeiss LSM710; Carl Zeiss AG, Jena, Germany) was used to analyze the FITC (Annexin V:FITC Apoptosis Detection kit, BD Biosciences, San Diego, CA) and PI fluorescent (Becton, Dickinson and Company, Franklin Lakes, NJ, USA) signals.

### Caspase-3 assay

A Caspase-3 Colorimetric Assay kit (KeyGen, Nanjing, China) was used to measure the caspase-3 activity according to the manufacturer’s instructions. Cells were cultured in serum-deprived α-MEM for 24 h to induce apoptosis, collected and washed with PBS. Briefly, cells were lysed on ice in lysis buffer for 1 h and vortexed every 20 min for 10 sec. This was followed by centrifugation for 1 min at 12,000 × g at 4°C. Aliquots of the supernatant containing 150 μg protein were diluted to 50 μl with cell lysis buffer, incubated with 5 μl of substrate at 37°C for 4 h in dark and a microplate reader (DNM-9602, Beijing Perlong Medical Instrument Ltd) was used to measure the absorbance value of the samples at 405 nm.

### Determination of the mitochondrial DNA (mtDNA) levels

qPCR was used to determine the relative amounts of mtDNA. qPCR (Taqman method) was performed in the Sequence Detection System 7500 (Applied Biosystems Life Technologies) following the manufacturer’s instructions. Briefly, a DNA extraction kit (Promega, Madison, WI, USA) was used to isolate the DNA from the cells on the tenth day of differentiation. Spectrophotometry at a wavelength of 260 nm was employed to quantify the DNA. A 110-nt mtDNA fragment within the CYTB gene was used to quantify mtDNA. A 291-bp region of the nuclear 28S gene was used to normalize the results. The ratio of mtDNA to nuclear DNA reflected the concentration of mitochondria per cell.

### Assessment of cellular ATP production

On the tenth day of differentiation, a luciferase-based luminescence assay kit (Beyotime, Nantong, China) was used to measure the ATP content of the P19 cells. Differentiated P19 cells were homogenized in an ice-cold ATP-releasing buffer and a single-tube luminometer (utrao SM600; Beyotime) was used to determine the ATP concentrations, which were normalized to the protein concentrations.

### Assessment of intracellular reactive oxygen species (ROS) levels

A 2′,7′-dichlorodihydrofluorescein diacetate acetyl ester (H_2_-DCFDA) probe (Beyotime) was used to estimate the intracellular ROS levels. The cells were incubated with 5 μM of H_2_-DCFDA for 30 min at 37°C, washed three times with pre-warmed PBS and then observed with a confocal laser-scanning microscope (excitation at a wavelength of 579 nm, emission at 644 nm, ×400 magnification; E4500; Nikon). Subsequently, the cells were trypsinized and centrifuged at 1,500 × g at room temperature for 5 min, washed twice with PBS, resuspended in PBS and analyzed by flow cytometry (Becton, Dickenson and Company).

### Antibodies and western blot analysis

Cultured cells were directly transferred to tubes containing lysis buffer and vortexed briefly. The supernatant was collected following centrifugation at 15,200 × g for 15 min at 4°C. Protein concentrations were determined using BCA protein assay reagent kit (KeyGen, Nanjing, China). Total proteins were isolated from cultured cells, separated on a 10% sodium dodecyl sulfate (SDS) gel by SDS polyacrylamide gel electrophoresis, and transferred onto polyvinylidene difluor-ide membranes. These membranes were incubated with a mouse polyclonal anti-WNT1, mouse monoclonal anti-GSK3β, rabbit polyclonal anti-β-catenin and mouse anti-β-actin antibody (Affinity, Santa Cruz, CA, USA), and goat anti-rabbit or rabbit anti-mouse immunoglobulin G–horseradish peroxidase conjugate (Amersham, UK). Immunoreactive proteins were detected by enhanced chemi-luminescence (Amersham, UK).

### Luciferase assay

The recombinant vector or pGL3-Basic vector (GenePharma, Shanghai, China) were cotransfected with the pRL-CMV vector (GenePharma) containing a *Renilla* luciferase reporter gene (as a normalizing control) into either the miR-19b knockdown or control stable P19 cells. The Dual Luciferase Reporter Assay system (Promega) was used to analyze the firefly and *Renilla* luciferase activities 36 h later.

### Statistical analysis

Each experiment was performed with at least 3 different cultures and repeated at least 3 times. Data are presented as the mean ± standard deviation (SD). For comparison of differences between groups, analysis of variance and unpaired Student’s t-tests were used. P<0.05 was considered to indicate a statistically significant difference.

## Results

### Transfection of P19 cells with the miR-19b knockdown vector

**Figure 1:**
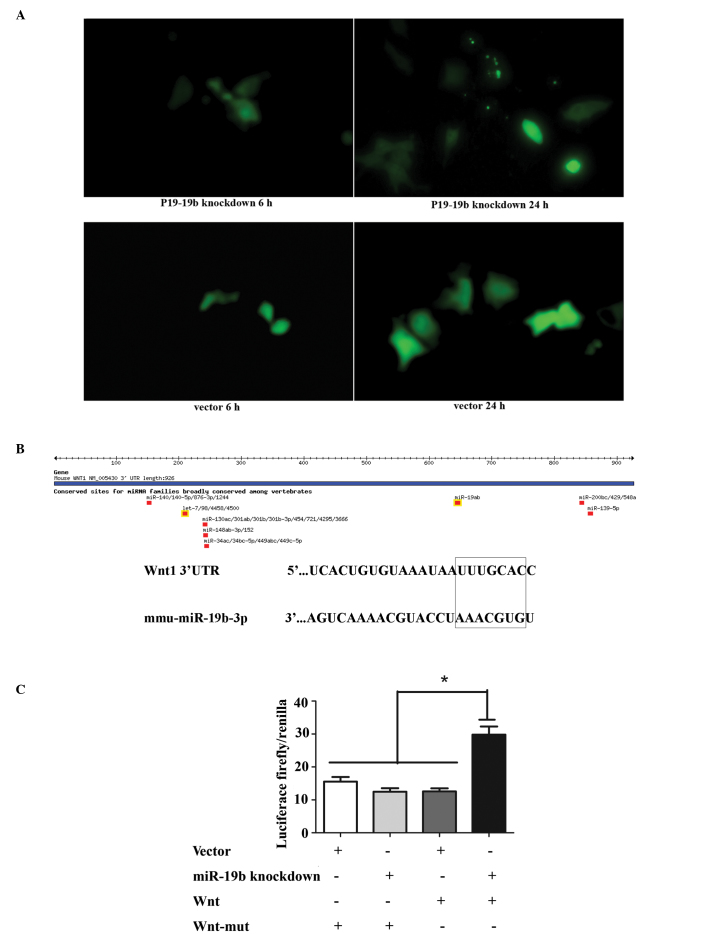
(A) Green fluorescent protein (GFP) expression. Fluorescence microscopy was used to observe the transfection efficiency, via GFP expression, of the microRNA-19b (miR-19b) knockdown vector and control vector in P19 cells. (B) Identification of miR-19b targets in the Wnt 3′ untranslated region. (C) The Dual-Luciferase^®^ Reporter Assay system was used to assess the luciferase activity. Data are presented as the mean ± standard error of the mean of three experiments (^*^P<0.05).

Plasmids pGLV3/H1/eGFP/Puro-miR-19b-3p-inhibitor sponge and pGLV3/H1/eGFP/Puro-miR-vector were transiently transfected into P19 cells. Observation of green fluorescent protein (GFP) expression under a fluorescence microscope indicated similar transfection efficiencies (Fig. 1A). Subsequently, stably transfected cells were selected by puromycin. Interaction between miRNAs and their target site(s) in the 3′ untranslated regions (3′-UTRs) results in translational repression or miRNA cleavage. Once the miRNAs are inhibited, the target gene becomes free from transcriptional repression and is activated, which can be detected by luciferase activity. In order to knockdown miR-19b (5′-UGUGCAAAUCCAUGCAAAACUGA-3′), complementary binding sites (5′-TCAGTTTTGCATGGATT TGCACA-3′) were inverted into the plasmid which were perfectly complementary with the sponge RNA (5′-GATCCT CAGTTTTGCATGGATTTGCACACTAGTCAGTTTTGCA TGGATTTGCACATTACCATCAGTTTTGCATGGATTTG CACAGAATTCAGTTTTGCATGGATTTGCACATTTTTT GAATT-3′). Previous studies have confirmed that the 3′-UTR of Wnt1 is a target of miR-19b. As expected, miR-19b knockdown significantly rescued the luciferase activity of the pGL3-wnt-3′-UTR reporter but not the mutated construct (mu-pGL3-Wnt-′3-UTR) (Fig. 1B and C; P<0.01). The result of the luciferase activity assay indirectly revealed that miR-19b was knocked down, demonstrating that the miR-19b-knockdown vector was constructed successfully.

### miR-19b knockdown inhibits cellular proliferation

**Figure 2:**
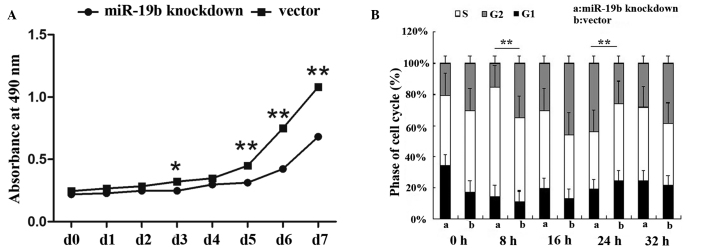
MicroRNA-19b (miR-19b) knockdown inhibits cell proliferation. (A) Cell proliferation. A cell counting kit-8 assay was used to monitor cell proliferation for seven consecutive days. (B) Cell cycle analysis. Cell cycle stages were monitored for each 8-h period. Results from both assays showed that miR-19b knockdown inhibited proliferation of P19 cells. Data are presented as the mean ± standard error of the mean of three experiments (^*^P<0.05, ^**^P<0.01).

The CCK-8 assay was used to assess the growth of miR-19b-knockdown and control P19 cells. At days 1, 2 and 4, the optical density (OD) values of the miR-19b-knockdown and control cells showed no significant differences. However, at days 5, 6 and 7, the OD values of the miR-19b-knockdown cells were significantly lower than those of the control cells. Thus, a reduced growth rate of the miR-19b-knockdown cells was observed compared with that observed in the control cells (Fig. 2A; P<0.05 and P<0.01). In addition, miR-19b-knockdown also affected the cell cycle. Flow cytometry of the cell cycle distribution detected a significantly lower percentage of miR-19b-knockdown P19 cells in the S phase of the cell cycle compared with that of the control cells (Fig. 2B; P<0.01).

### MiR-19b knockdown reduces the levels of apoptosis in P19 cells

**Figure 3:**
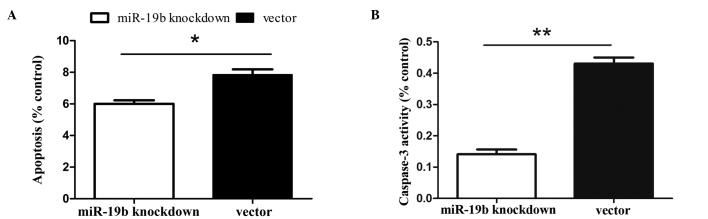
The effect of the microRNA-19b (miR-19b) knockdown on cellular apoptosis. (A) Binding of Annexin V-fluorescein isothiocyanate to detect cell apoptosis. (B) Cell apoptosis detected with caspase-3 activity. These results indicate that miR-19b knockdown inhibits serum deprivation-induced apoptosis. Data are presented as the mean ± standard error of the mean of three experiments (^*^P<0.05, ^**^P<0.01).

Caspase-3 protein assays and Annexin V-FITC, which binds to phosphatidylserine, were used to detect the early stages of apoptosis. Cells were induced to undergo apoptosis via 24 h of serum starvation. Binding experiments with Annexin V-FITC indicated that miR-19b knockdown reduced the number of apoptotic cells in response to serum deprivation (Fig. 3A; P<0.05). Additionally, the caspase-3 activity assay (Fig. 3B; P<0.01) revealed that miR-19b knockdown reduced the number of apoptotic cells in response to serum deprivation. These results indicate that miR-19b knockdown inhibits serum deprivation-induced apoptosis in P19 cells.

### Effects of miR-19b knockdown on mtDNA copy number in P19 cells

**Figure 4:**
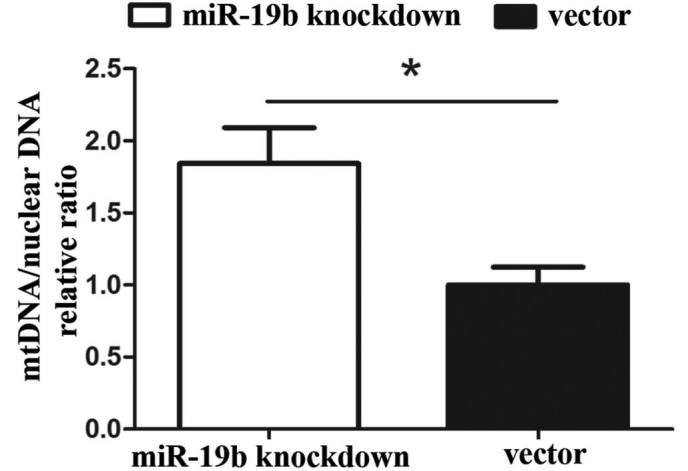
Assessment of mitochondrial DNA (mtDNA) content. The results show a significantly higher mtDNA copy number in the microRNA-19b (miR-19b) knockdown group compared with that in the vector group. Data are presented as the mean ± standard error of the mean of three experiments (^*^P<0.05).

The relative expression levels of mitochondria were assessed, as these represent the total mtDNA copy number in the two cell lines. The mtDNA copy number per mitochondrion is considered to be constant in all mammalian cell types. As a result, the copy number of mtDNA can be determined from the relative quantity of mitochondria. qPCR revealed that the mtDNA copy number was significantly higher in the miR-19b knockdown group compared with that in the vector group (Fig. 4; P<0.05).

### Cellular ATP production increases upon miR-19b knockdown

**Figure 5:**
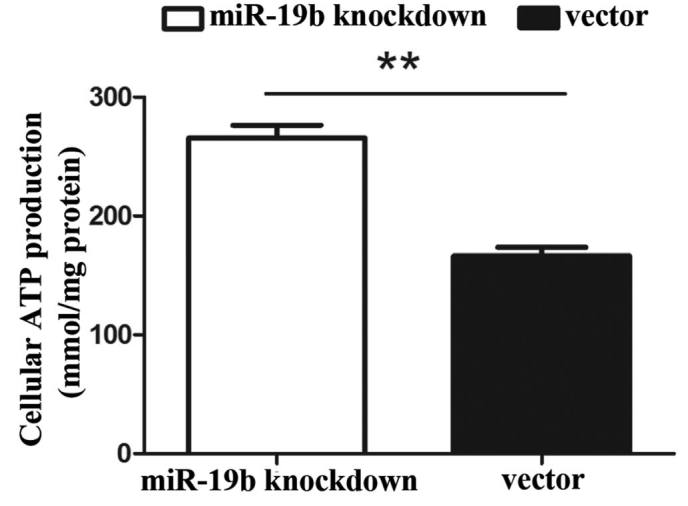
Effects of microRNA-19b (miR-19b) knockdown on intracellular ATP levels. Total cellular ATP production was increased in the miR-19b knockdown cells. Data are presented as the mean ± standard error of the mean of three experiments (^**^P<0.01).

In eukaryotic cells, the mitochondrion is the major platform for energy transduction, producing ATP via the oxidative metabolism of nutrients. Impaired mitochondria may lead to a reduction in the levels of ATP. The results of the current study revealed that the total cellular production levels of ATP were increased in the miR-19b knockdown cells compared with those in the control cells (Fig. 5; P<0.01).

### Effects of miR-19b knockdown on intracellular ROS levels

**Figure 6:**
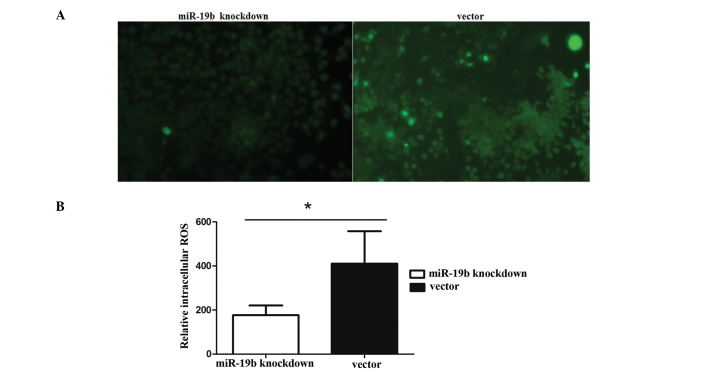
Effects of microRNA-19b (miR-19b) knockdown on intracellular reactive oxygen species (ROS) content in differentiated cells. (A) The fluorescence of 2′,7′-dichlorodihydrofluorescein diacetate was used to determine the ROS levels, using a confocal laser-scanning microscope. (B) Fluorescence-activated cell sorting analysis determination of ROS levels. Data are presented as the mean ± standard error of the mean of three experiments (^*^P<0.05).

Subsequently, the effect of miR-19b knockdown on the ROS content of cells was investigated. The levels of ROS in the miR-19b-knockdown cells were much lower than those in the control cells (Fig. 6A), as indicated by less intense fluorescent signals in the presence of the H_2_-DCFDA (Fig. 6B). Hence, the results clearly demonstrate that miR-19b knockdown inhibits apoptosis, accompanied by mitochondrial dysfunction in P19 cells.

### MiR-19b knockdown has no clear effect on the morphology of P19 cells during differentiation

**Figure 7:**
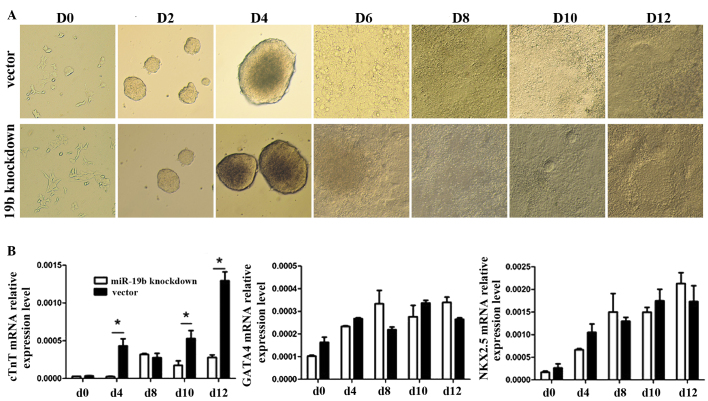
Effect of microRNA-19b (miR-19b) knockdown on the differentiation of P19 cells. (A) Morphological appearance of differentiating P19 cells. P19 cells (miR-19b-knockdown and negative control groups) were stimulated to differentiate for ~12 days. On days 0, 2, 4, 6, 8, 10 and 12, images of the cells were captured. (B) Expression levels of myocardial cell molecular markers at various time points during the induction of differentiation (days 0, 4, 8, 10 and 12). Significant differences in the expression levels of these markers were found between the miR-19b knockdown and negative control groups. Data are presented as the mean ± standard error of the mean of six experiments (^*^P<0.05).

To investigate the ability of the miR-19b-knockdown P19 cells to differentiate into myocardial cells, the morphological appearance was observed and myocardial-specific molecular markers (cTnT, GATA4 and NKX2.5) were quantified, and the appearance of beating cell clusters during DMSO-induced differentiation was monitored (Fig. 7A). However, no differences were observed in the cell morphology or the time taken for the appearance of beating cell clusters between the miR-19b-knockdown cells and the control cells. In addition to observing cell morphology, the expression of myocardial-specific molecular markers was analyzed at the RNA level, including cTnI, GATA4 and NKX2.5, which are known to have upregulated levels of expression during the differentiation of mouse P19 cells into myocardial cells (Fig. 7B; P<0.05). The expression levels of these marker genes were detected by RT-qPCR in the two cell lines on days 0, 4, 8, 10 and 12. Expression levels of all the marker genes gradually increased during the process of differentiation, however, in the miR-19b-knockdown cells only cTnT showed significantly lower expression levels compared with those observed in the control cells at days 4, 10 and 12.

### Effect of miR-19b knockdown on the Wnt/β-catenin signaling pathway

**Figure 8:**
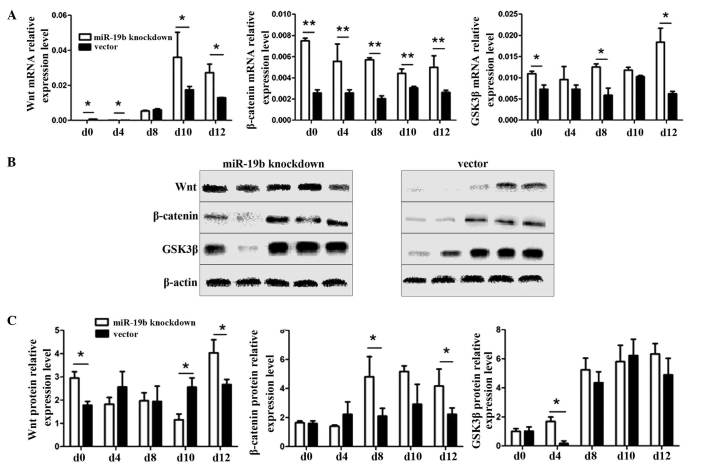
Effect of microRNA-19b (miR-19b) on the Wnt/β-catenin signaling pathway. (A) mRNA and (B and C) protein levels of molecules in the Wnt/β-catenin signaling pathway (WNT, β-catenin and GSK3β) at various time points during the stimulation of differentiation. Data are presented as the mean ± standard error of the mean of three experiments (^*^P<0.05; ^**^P<0.01).

Wnt is an essential regulator of cell differentiation. The Wnt protein initiates the Wnt/β-catenin signaling pathway, GSK3β acts as a switch and β-catenin functions as the effector molecule. Therefore, qPCR and western blot analyses were used to detect the RNA and protein expression changes of several key molecules in the Wnt signaling pathway (Wnt, GSK3β and β-catenin). During the induction of differentiation of P19 cells into myocardial cells, the relative expression levels of Wnt, GSK3β and β-catenin were significantly higher compared with those in the vector group at almost all time points (Fig. 8A; P<0.05 and P<0.01). At the protein level, the expression levels of Wnt in the miR-19b-knockdown group were significantly higher than those in the vector group at days 0, 10 and 12, those of β-catenin were significantly higher than those in the vector group at days 8 and 12, those of GSK3β were significantly higher than those in the vector group at day 4, and the trend in the levels of β-catenin expression was similar to that of Wnt1 (Fig. 8B and C, P<0.05).

## Discussion

MiRNAs contribute to cardiac development and can be used as novel biomarkers and therapeutic targets for CHDs (2,14). Previous studies have determined that the miR-17–92 cluster, which includes mir-19b, is important in a number of diseases and miR-19b expression may correlate with the incidence of cardiovascular diseases and cardiogenesis (15). Gao *et al* (16) found that the downregulation of miR-19b contributes to angiotensin II-induced overexpression of connective tissue growth factor in cardiomyocytes. Jung *et al* (17) determined that there is an interaction between the hepatitis B virus (HBV) and the miR-17–92 polycistron via c-Myc, and that miR-20a and miR-92a-1 induce post-transcriptional suppression of HBV. In the present study, the P19 cell line was used as a research model, and a stable line of miR-19b-knockdown P19 cells was established to evaluate the effect of this miRNA on P19 cells and their differentiation toward myocardial cells. MiR-19b knockdown inhibited proliferation and apoptosis in the P19 cells. Notably, a previous study found that the overexpression of miR-19b could increase proliferation, inhibit apoptosis and promote differentiation of P19 cells into mature cardiac cells (18). This indicates that overexpression of miR-19b or miR-19b knockdown may influence morphogenesis in the embryonic heart by inhibiting excessive apoptosis in the myocardium. Embryonic fetal heart growth depends on the balance between cardiomyocyte proliferation and apoptosis (19). Inadequate proliferation or excess apoptosis may directly or indirectly result in CHD (20), which is often caused by altered proliferation and/or apoptosis in the septum, neighboring tissue or myocardium (21).

The results of the present study, including those from the CCK-8 assay and cell cycle analysis, indicate that miR-19b knockdown inhibits the proliferation of P19 cells by significantly reducing the percentage of cells in the S phase, however, the specific mechanism by which this occurs remains to be determined. Yan *et al* (22) determined that overexpression of the miR-17–92 cluster markedly inhibited hypoxia-induced apoptosis. Sharifi *et al* (23) found that inhibition of miR-92a inhibited cell proliferation in human acute promyelocytic leukemia. In the current study, miR-19b knockdown was determined to significantly inhibit serum starvation-induced apoptosis. The molecular mechanism that underlies this effect remains unknown. Crow *et al* (24) hypothesized that mitochondria are important in the transmission and amplification of apoptotic signals. Mitochondria are at the center of the regulatory processes for apoptosis (25). In the present study, it was demonstrated that levels of intracellular ROS were reduced, ATP contents were increased and the levels of mitochondrial DNA were increased on the tenth day of differentiation of P19 cells. It may be hypothesized that miR-19b knockdown cells generate more ATP to compensate for lost ATP production, which correlates with the increased level of mitochondrial DNA. It was observed that miR-19b knockdown does not significantly affect the differentiation of P19 cells into cardiomyocytes, indicated by the lack of morphological changes and the normal expression of cardiomyogenesis-specific molecular markers. Although the expression of all the marker genes (cTnT, GATA4 and NKX2.5) gradually increased during differentiation, only cTnT in miR-19b-knockdown cells showed significantly lower expression levels than those in the control cells at days 4, 10 and 12.

Furthermore, the results of the present study demonstrate that miR-19b does affect the Wnt/β-catenin signaling pathway. Wnt signaling is an essential regulator of cardiovascular differentiation, morphogenesis and progenitor self-renewal (26). Given that miRNAs negatively regulate their targets, miR-19b knockdown should upregulate its potential targets. A previous study revealed that miR-19b may indirectly target Wnt1 mRNA through its 3′-UTR (16). In the present study, the results of the luciferase assay indicate that miR-19b knockdown rescued Wnt1 expression by removing its interaction with its cognate miRNA. Wnts were initially considered suppressive of heart formation. Wnts 1, 3A and 8 act via the inhibition of GSK3, allowing nuclear localization of β-catenin, which appears to inhibit cardiac differentiation, whereas the non-canonical Wnt11, along with protein kinase C, appears to enhance cardiac differentiation (27). Previous results in zebrafish indicate that β-catenin signaling is blocked in heart valve formation, which demonstrates a negative role of Wnts in heart development (28). In the present study, miR-19b knockdown affected differentiation by increasing the activation of the Wnt/β-catenin signaling pathway via an essential upstream target of Wnt1.

Furthermore, Wnt/β-catenin signaling can promote cardiogenesis by inducing the proliferation of cardiac progenitor cells in the secondary heart field (29–31). Wnt/β-catenin signaling is also important in the endocardium, where it regulates the specification and proliferation of endocardial cushion cells (32). In the present study, following the successful knockdown of miR-19b, which normally acts on the 3′-UTR of Wnt1, the levels of Wnt1 protein expression significantly increased, thereby activating Wnt/β-catenin signaling and inhibiting myocardial cell development.

Previous studies indicate that endogenous miR-19b may have a key regulatory role in constraining the production of pro-inflammatory cytokines and chemokines by fibroblast-like synoviocytes and hence contribute to the pathology of inflammation (33). MiR-19b is also a novel regulator of fibrotic TGF-β signaling and the loss of miR-19b following hepatic stellate cell (HSC) activation perpetuates the fibrotic response (34). Furthermore, the miR-19a/b family regulates cardiac hypertrophy and survival by repressing the target genes atrogin-1 and MuRF-1 (35).

In conclusion, the results of the present study demonstrate that miR-19b knockdown significantly inhibits the proliferation and apoptosis of P19 cells. MiR-19b knockdown results in an increase in Wnt expression levels, which activates the Wnt/β-catenin signaling pathway in P19 cells, and may regulate the cardiomyocyte differentiation of P19 cells. These results indicate that miR-19b overexpression and knockdown leads to an imbalance between proliferation and apoptosis, which may result in embryonic cardiac malformations. This study of miR-19b provides insight into novel therapeutic strategies for CHD. Further study into the functions of related miRNAs may elucidate the processes of pathogenesis during cardiogenesis. However, the molecular mechanisms that mediate the balance between proliferation and apoptosis in response to miR-19b knockdown or overexpression require further investigation.
